# Potentially Inappropriate Medications among Elderly with Frailty in a Tertiary Care Academic Medical Centre in Saudi Arabia

**DOI:** 10.3390/healthcare10081440

**Published:** 2022-07-31

**Authors:** Saad Mohammad Alsaad, Sheikah AlEraij, Abdulaziz Mohammed Alsaad, Haytham Ibrahim AlSaif, Ghada Bawazeer

**Affiliations:** 1 Department of Family and Community Medicine, College of Medicine, King Saud University, Riyadh 11462, Saudi Arabia; hayalsaif@ksu.edu.sa; 2 Postgraduate Residency Training Program, Family and Community Medicine Department, King Saud University Medical City, Riyadh 11462, Saudi Arabia; aleraij.sh@gmail.com; 3 Medical Intern, College of Medicine, King Saud bin Abdulaziz University for Health Sciences, Riyadh 14611, Saudi Arabia; aziz.fnais@gmail.com; 4 Clinical Pharmacy Department, College of Pharmacy, King Saud University, Riyadh 11362, Saudi Arabia; gbawazeer@ksu.edu.sa

**Keywords:** frailty, frail elderly, polypharmacy, potentially inappropriate medication, Saudi Arabia

## Abstract

This study aims to assess the prevalence of potentially inappropriate medications (PIMs) and to analyze the relationship between the PIMs and frailty among inpatient older adults aged 65 and above in Saudi Arabia. A retrospective cross-sectional study design was utilized during the period between April 2021 and April 2022 of all patients aged 65 years and above admitted in a public tertiary hospital in Saudi Arabia. Data on the number of medications and the use of PIMs were assessed using Beers’ criteria while the frailty status was assessed using the “FRAIL Scale”. Of the 358 patient files that were reviewed, 52.2% were males, 60.9% were aged 65–74 years, and 82% were married. The prevalence of robust, prefrail, and frail patients was 5%, 36.9%, and 58.1%, respectively. According to the 2019 Beers criteria, a total of 45.8% (n = 164) participants identified as using PIMs. Compared to the non-PIMs group, the PIMs group demonstrated significant differences in the number of medications (*p* < 0.001), the number of comorbidities (*p* < 0.05), and the frailty score (*p* < 0.001). The strongest predictor of PIM use was a number of comorbidities, recording an odds ratio of 2.86, (95% CI 1.21–6.77, *p* < 0.05). Our results show that the use of PIM was significantly associated with frail older adults with multiple comorbidities and in patients with polypharmacy. A clear assessment and evaluation tool may improve the quality of drug treatment in the older adult population, particularly in frail patients.

## 1. Introduction

The demographic trend of the elderly in Saudi Arabia follows the global increase and will considerably rise over the next few decades. According to the United Nations, the population aged 65 or older in Saudi Arabia is estimated to reach 18.4% (10 million) by 2050 [1]. The rise of the elderly population increases the morbidity burden in the healthcare system [2]. Older adults are more likely to be characterized by multiple comorbidities or with two or more chronic conditions and aging-related conditions (e.g., frailty) that need continuous care [3,4,5]. Older people who suffer from multiple comorbidities require prescriptions from several physicians and specialists involved in the patient care.

Polypharmacy is the administration of multiple (five or more) medications that are common in elderly patients [6,7]. Polypharmacy has been associated with a high risk of adverse drug reactions and adverse drug-drug and drug-disease interactions [7] in patients. Older individuals are vulnerable to adverse drug reactions due to physiological changes, genetic predisposition, and environmental exposure [7,8]. Prescriptions that pose a high risk of adverse reaction and that should be used with caution for older individuals are referred to as potentially inappropriate medication (PIM) [9]. 

Several tools and strategies have recently been developed to identify PIM use in older individuals. Among these tools that identify PIM uses were the American Geriatrics Society (AGS) Beers Criteria, the Screening Tool of Older Persons’ Potentially Inappropriate Prescriptions (STOPP), and the Screening Tool to Alert Doctors to the Right Treatment (START) [10,11,12,13]. The Beers’ criteria are one of the most common tools for PIM use in older adults, which also serve as guidelines for healthcare professionals to help improve the safety of prescribing medications [10,11]. Several countries’ studies have evaluated the prevalence of PIM use in the elderly using the Beers criteria, such as the United States, China, India, and other developing countries [14,15,16,17].

Moreover, several studies have shown that older adults who are more likely to have frailty are more likely to receive multiple prescriptions which increases the risk of receiving PIMs [18,19,20]. Frail patients who are receiving multiple prescriptions and PIM use are vulnerable to various risks such as adverse events from both interactions (drug–drug) and contraindications (drug–disease) [20]. The concept of frailty as one of the serious public health concerns in the geriatric population is now well recognized worldwide, including in Saudi Arabia [21]. Frailty is defined as a clinical syndrome of physiological vulnerability and a high risk of adverse health outcomes [22]. Screening and evaluation for frailty are encouraged for physicians to develop a personalized care plan [23]. This fact emphasizes the need for further studies regarding the relationship of these variables with frailty syndrome. The identification of the association of variables could lead to the improvement of preventive clinical approaches among older people and management of frailty syndrome. This study aims to assess the prevalence of PIMs and analyze the relationship between the PIMs and frailty among inpatient older adults aged 65 and above who are admitted to King Saud University Medical City (KSUMC). 

## 2. Materials and Methods

### 2.1. Study Setting and Participants

A retrospective cross-sectional study design was used. The study utilized the electronic medical records (EMRs) of all patients aged 65 years and older admitted to KSUMC during the period between April 2021 and April 2022. We assumed a 5% margin of error and a confidence level of 95% based on previous literature, a formula calculated sample size for a single proportion and produced a minimum number of 358 patients to be included. The inclusion criteria were: 65 years and older, admitted to KSUMC medical wards. Exclusion criteria were: patients diagnosed with cognitive impairment, terminal illness, on palliative care, patients with incomplete medical records, and those with 1-day admissions. 

### 2.2. Measures

Demographic data (age, gender, body mass index (BMI), comorbidities, self-reported subjective health, history of falls, and history of hospital admission in the past 3 and 6 months were collected from medical records. Data on the number of medications and the use of PIM were assessed using Beers’ criteria. The frailty status was assessed using the “FRAIL Scale” [24]. The FRAIL Score ranges from 0 to 5, where 0 = best, 5 = worst, and represents frail (3 to 5), pre-frail (1 to 2), and robust (0) health status.

### 2.3. Statistical Analysis

Data analysis was performed using IBM SPSS Statistics v25.0. We used descriptive analysis for all categorical variables and then reported in terms of numbers and percentages. The Chi-square test was used to determine the association with frailty among PIM patients and non-PIM patients. Logistic regression analysis was used to identify the association between frailty score and other demographic variables. A *p*-value of <0.05 was considered to show a statistically significant difference. 

### 2.4. Ethical Considerations

Researchers provided complete confidentiality and anonymity by collecting information that avoided identifying information. The study was approved on 28 April 2021 by the Institutional Review Board, college of medicine, King Saud University (project number E-21-5912).

## 3. Results

**Figure 1:**
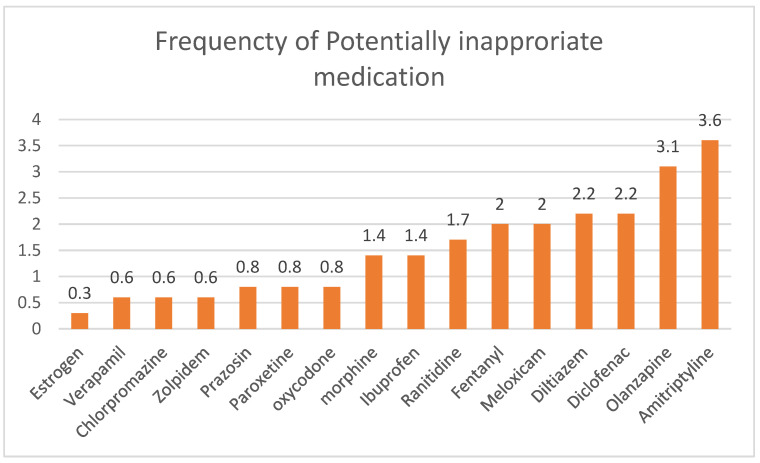
Percentage of PIM prescribed to study participants based on the Beers criteria.

During the study period, 358 patient files were reviewed. The study population was composed of 52.2% males and 47.8% females. Of the 358 participants, 60.9% were aged 65–74 years, 82% were married, and most had no formal education (*n* = 257, 71.8%). Nearly half of the participants had poor self-reported subjective health (*n* = 144, 45.3%), and 32.2% had a BMI of greater than 30 kg/m^2^. Thirty-six percent of the participants experienced a fall one or more times. Most participants had ten or more medications (*n* = 286, 80.3%) and two or more comorbidities (*n* = 332, 92.7%). The prevalence of robust, prefrail, and frail patients were 5, 36.9, and 58.1%, respectively. Figure 1 shows the most common PIMs prescribed to study participants based on the Beers criteria. The prevalence of robust, pre-frail and frail patients was 5, 36.9, and 58.1%, respectively. Detailed demographic characteristics of the participants are presented in Table 1.

According to the 2019 Beers criteria, a total of 45.8% (*n* = 164) of participants identified as using PIM. More than half of the PIM group was aged between 65 and 74 years (58.5%) (Table 1). The majority of the participants who used PIM were married (81.1%), had no formal education (74.4%), had less than two hospital admissions in the previous 3 (97%) and 6 months (95.7%), had ten or more medications (89%), and had two or more comorbidities (89%). Details of the PIMs identified in this study are presented in Table 1. Compared to the non-PIM group, the PIM group demonstrated significant differences in the number of medications (*p* < 0.001), the number of comorbidities (*p* < 0.05), and the frailty score (*p* < 0.001).

Table 2 shows the results of the association between demographic factors and PIM use using logistic regression analysis. As shown in Table 2, only five independent variables (number of hospital admission in the previous three and six months, number of medications, number of comorbidities, and frailty score) made a unique statistically significant association with PIM use in patients. The strongest predictor of PIM use was a number of comorbidities, recording an odds ratio of 2.86. This indicated that participants with two or more comorbidities were two times more likely to use PIM than those with less than two or no comorbidities (95% CI: 1.21–6.77, *p* < 0.05). No significant association was found between other demographic variables. 

## 4. Discussion

This study examined the prevalence of PIMs, the relationship between frailty among inpatient older adults aged 65 and older, and associated factors. Our findings show that most participants had ten or more medications (80.3%) and two or more comorbidities (92.7%), with 45.8% of the participants being exposed to PIM. The findings also show that the number of hospital admissions in the previous three and six months, the number of medications, the number of comorbidities, and the frailty score had a significant association with PIM use in patients. Identifying these patient factors may be a precautionary measure before prescribing medication to elderly patients.

The prevalence of polypharmacy in the present study is similar to studies conducted in China, Portugal, Sweden, and the United States [25,26,27,28]. In addition, excessive polypharmacy that was reported in this study was comparable in elderly inpatients in China [15]. Excessive polypharmacy (10 or more medications) is markedly prevalent in this sample of participants. The number of prescribed medications varied across countries. One could think that the difference is based on physicians’ attitudes toward treatments for severe or complex patients [29,30]. The number of comorbidities of patients may drive the high prevalence of excessive polypharmacy. The present study identified that most participants had two or more comorbidities, which may explain the prevalence.

This study also highlights our study’s results, indicating that the prevalence of frailty in people aged 60 years or older in our cohort is 58.1%. This is higher compared to the previous study among Saudi community-dwelling older adults [31]. On the other hand, the finding is similar to a study conducted in Cuba that found that the prevalence rate of 51% in the elderly [32]. Several epidemiological studies found an association between increasing age in the prevalence of frailty [33,34,35]. Such factors could trigger polypharmacy and the use of PIMs in older patients. A previous study had highlighted that frailty leads to a need for additional medications resulting in polypharmacy in older adults [36]. 

The present study found an association between the PIMs and in patients with two or more comorbidities, polypharmacy, and frail patients. Previous studies found an association between polypharmacy and frailty for people 65 years and older [33,34]. Our findings are in line with the previous studies that found patients with polypharmacy and PIMs were more likely to be frail and have multiple comorbidities [37,38]. The results also reveal that no significant relationship between age, BMI, falls, and self-reported subjective health and PIM. This result is surprising considering the increasing age was influenced by PIMs. This is contrary to previous studies that found that PIMs influenced subsequent falls and increased age [39,40]. However, a study in the United States shows a mixed results between prescribing PIMs and age [41]. The study revealed no significant association between prescribing PIMs and adults aged 75–84 [41]. Further studies are needed about the effect of medical optimization interventions on clinical outcomes among older adults. In addition, physicians may need to include a comprehensive evaluation of patients with these factors to go through the medication process or use of PIMs. Furthermore, a comprehensive medication assessment could be helpful and should be considered during the patient’s visit to confirm the indication (e.g., medication–condition matching), dosage (e.g., dosages appropriate for renal and/or liver function), duration, adverse effects, and patient’s health literacy. 

Our study acknowledges some limitations. First, we had a small sample size of age 65 and older, which limits the generalizability. Second, the cross-sectional design of our study does not provide evidence of cause-and-effect between the variables. Nevertheless, this study provides additional knowledge on polypharmacy and the use of PIMs among frail patients in Saudi Arabia. 

## 5. Conclusions

Our results show that the use of PIM was significantly associated with frail older adults with multiple comorbidities and in patients with polypharmacy. However, this study showed a need for further study with a longitudinal nature to assess the causality of these conditions with frailty. The goals of medication management among older patients include reducing adverse drug reactions and eliminating duplication, as well as improved patient adherence. A clear assessment and evaluation tool may improve the quality and measure of drug treatment in the older adult population. Future studies are also needed to focus on different components (e.g., clinical outcomes such as mortality and cognitive impairment) for evaluating the quality of drug treatment among patients with multiple comorbidities and frail older patients. 

## Figures and Tables

**Table 1:** Demographic characteristics identified based on the Beers criteria.

Characteristics	Overall (*n* = 358)	PIM (*n* = 164)	Non-PIM (*n* = 194)	*p*-Value
**Age**				0.415
From 65 to 74 years	218 (60.9)	96 (58.5)	122 (62.9)	
From 75 to 84 years	108 (30.2)	55 (33.5)	53 (27.3)	
More than 85	32 (8.9)	13 (7.9)	19 (9.8)	
**Gender**				0.188
Male	187 (52.2)	81 (49.4)	106 (54.6)	
Female	171 (47.8)	83 (50.6)	88 (45.4)	
**Marital status**				0.371
Single	64 (17.9)	31 (18.9)	33 (17.0)	
Married	294 (82.1)	133 (81.1)	161 (83.0)	
**Education level**				0.319
Non	257 (71.8)	122 (74.4)	135 (69.6)	
Primary	17 (4.7)	10 (6.1)	7 (3.6)	
Secondary	46 (12.8)	17 (10.4)	29 (14.9)	
Tertiary	38 (10.6)	15 (9.1)	23 (11.9)	
**Self-reported subjective health**				0.938
Poor	162 (45.3)	75 (45.7)	87 (44.9)	
Moderate	115 (32.1)	51 (31.1)	64 (32.9)	
Good	81 (22.6)	38 (23.2)	43 (22.2)	
**BMI**				0.574
Underweight	6 (1.7)	4 (2.4)	2 (1.0)	
Normal weight	99 (27.7)	49 (29.9)	50 (25.8)	
Overweight	115 (32.1)	51 (31.1)	64 (33.0)	
Obesity	138 (38.5)	60(36.6)	78 (40.2)	
**Number of hospital admission in previous 3 months**				0.397
Less than 2	349 (97.5)	159 (97.0)	190 (97.9)	
2 or more	9 (2.5)	5 (3.0)	4 (2.1)	
**Number of hospital admission in previous 6 months**				0.127
Less than 2	336 (93.9)	157 (95.7)	179 (92.3)	
2 or more	22 (6.1)	7 (4.3)	15 (7.7)	
**Number of times fall experienced in previous 1 month**				0.052
None	225 (62.8)	92 (56.1)	133 (68.6)	
One or more	133 (37.2)	71 (43.3)	62 (31.4)	
**Number of medications**				**0.001**
From 1 to 4	12 (3.4)	1 (0.6)	11 (5.7)	
From 5 to 9 (polypharmacy)	58 (16.3)	17 (10.4)	41 (21.2)	
10 or more (excessive polypharmacy)	288 (80.4)	146 (89.0)	142 (73.1)	
**Number of comorbidities**				**0.041**
No comorbidity	5 (1.4)	3 (1.8)	2 (1.0)	
One	21 (5.9)	15 (9.1)	6 (3.1)	
Two or more	332 (92.7)	146 (89.0)	186 (95.9)	
**Frailty score**				**0.009**
Robust	18 (5.0)	2 (1.2)	16 (18.2)	
Pre-frail	132 (36.9)	61 (37.2)	71 (36.6)	
Frail	208 (58.1)	101 (61.6)	107 (55.2)	

Note: PIM = potentially inappropriate medication; Statistically associated at 0.05 level of significance.

**Table 2:** Association between demographic factors and potentially inappropriate medication use using logistic regression analysis.

Characteristics	OR	Wald	95% CI	*p*-Value
**Age**				0.345
From 65 to 74 years	Ref			
From 75 to 84 years	0.78	0.71	0.44–1.37	
More than 85	1.64	0.83	0.61–3.76	
**Gender**				0.508
Male	Ref			
Female	0.83	0.43	0.48–1.42	
**Marital status**				0.841
Single	Ref			
Married	0.98	0.03	0.51–1.85	
**Education level**				0.593
Non	Ref			
Primary	0.87	0.70	0.22–2.15	
Secondary	1.25	1.61	0.71–3.64	
Tertiary	0.94	1.15	0.47–2.81	
**Self-reported subjective health**				0.783
Poor	Ref			
Moderate	0.90	0.12	0.52–1.56	
Good	0.80	0.48	0.42–1.49	
**BMI**				0.715
Normal weight	Ref			
Underweight	0.58	0.36	0.10–3.39	
Overweight	1.13	0.19	0.64–1.19	
Obesity	1.26	0.77	0.74–2.15	
**Number of hospital admissions in previous 3 months**				**0.046**
Less than 2	Ref			
2 or more	0.11	3.98	0.01–0.96	
**Number of hospital admissions in previous 6 months**				**0.044**
Less than 2	Ref			
2 or more	1.87	1.43	1.09–3.47	
**Number of falls experienced in previous 1 month**				0.089
None	Ref			
One or more	0.64	2.89	0.38–1.06	
**Number of medications (polypharmacy)**	0.36	14.32	0.22–0.61	**0.001**
**Number of comorbidities**	2.86	5.72	1.21–6.77	**0.016**
**Frailty score**				**0.030**
Pre-frail	Ref			
Robust	0.13	6.26	0.30–0.65	
Frail	0.14	6.22	0.32– 0.65	

Note: CI = confidence interval; PIM = potentially inappropriate medication; Statistically associated at 0.05 level of significance.

## Data Availability

The data set used is locked and stored in the College of Medicine at King Saud University and can be obtained from the principal investigator upon reasonable request.
